# A new strategy of enteral nutrition intervention for ICU patients targeting intestinal flora

**DOI:** 10.1097/MD.0000000000027763

**Published:** 2021-11-24

**Authors:** Yangyang Guo, Ming Xu, Guangzhi Shi, Jindong Zhang

**Affiliations:** a Intensive Care Unit, Beijing Tiantan Hospital, Capital Medical University, Beijing, China; b Department of Gastroenterology, Peking University Third Hospital, Beijing, China.

**Keywords:** enteral nutrition, gut microbiota, intensive care unit, microbial marker

## Abstract

**Background:**

Enteral nutrition (EN) therapy is a routine supportive method for patients in the intensive care unit (ICU). However, the incidence of EN intolerance is prevalent, because most ICU patients suffer intestinal mucosal barrier damage and gastrointestinal motility disorder. There is no definite index to predict EN intolerance, and the current treatment methods are not effective in alleviating EN intolerance. Gut microbiota is an important component of the intestinal micro-ecological environment, and alterations in its structure and composition can reflect changes in intestinal function and microenvironment. The purpose of this study is to investigate the effect of EN on the gut microbiota of ICU patients by monitoring the dynamic alterations of gut microbiota and to screen out the microbial markers that can be used to predict the incidence of EN intolerance.

**Methods:**

One hundred ICU patients with trauma or in a period of acute stress after surgery will be enrolled, and their fecal samples will be collected at different timepoints for microbial sequencing and analysis. General clinical data (demographic information, surgical data, laboratory parameters, illness severity scores, and therapeutic drugs), nutritional status data (nutritional status assessment and nutrition therapy monitoring data), as well as clinical outcomes, will be recorded. The microbial and clinical data will be combined to analyze the baseline characteristics and dynamic alterations of gut microbiota along with the incidence of EN intolerance. Data related to the gut microbiota will be statistically analyzed by R software, and other data performed by SPSS23.0 software.

**Conclusions:**

The effect of EN on gut microbiota and microbial markers predicting the intolerance of EN will lead us to develop a new nutrition intervention strategy for ICU patients. Furthermore, the results of this study will provide a basis for the discovery of potential probiotics used for the prevention and treatment of EN intolerance.

## 1 Introduction

Nutritional status is an important factor that directly affects the outcome of intensive care unit (ICU) patients and is closely related to the survival of ICU patients.^[1]^ At present, nutritional supportive approaches used in clinical practice include enteral nutrition (EN) and parenteral nutrition,^[2]^ and the former has advantages in maintaining the integrity of the intestinal barrier, preventing intestinal bacterial translocation, and reducing enterogenous infection. In addition, EN is more fit in with human physiology than parenteral nutrition and plays an irreplaceable role in the secretion and motility functions of the gastrointestinal tract.^[3–5]^

Early EN is widely accepted and used in the field of critical care medicine. However, for ICU patients, the implementation of EN is accompanied by great risks and challenges. The incidence rate of EN intolerance is very high. Severe trauma, shock, and postoperative critical patients are difficult to tolerate EN, and very few even occur nonobstructive intestinal necrosis. Retrospective analysis of nutrition practice data from 1888 patients in 167 ICUs worldwide found that the incidence rate of interruption of feeding due to EN intolerance was as high as 30.5% (576/1888), and clinical symptoms of EN intolerance appeared on average within 3 days (range: 1–12 days).^[6]^ For ICU patients, the need for nutrition is urgent, and if the application of EN is blocked due to intolerance, it will cause a second hit to the patients and hurt the prognosis of the patients. Therefore, it is an urgent task to reveal the causes of EN intolerance in ICU patients under stress state and to find effective treatment methods.

Although many researchers have tried to explore the causes of EN intolerance from the demographic or clinical characteristics, no appropriate laboratory or clinical indicators have been found to guide the nutritional supportive treatment.^[7–12]^ Gungabissoon U et al^[6]^ conducted demographic statistics on patients with EN tolerance and intolerance in ICU, and found that there were no statistical differences in age, gender, body mass index (BMI), and disease severity between the 2 groups. Lavrentieva A et al^[13]^ also found that there were no statistical differences in age, sex, burn index, burn area percentage, Acute Physiology, and Chronic Health Evaluation II score and sequential organ failure assessment score between septic burn patients with EN tolerance and intolerance.

Due to the unclear mechanism, there is still a lack of objective indicators for the evaluation and monitoring of EN intolerance. The accuracy and reliability of judging EN tolerance of critically ill patients based on clinical symptoms are limited. Gastrointestinal motility and gastric residual volume can be determined by ultrasound or gastrointestinal drainage volume, which is also a monitoring method. However, there is still no conclusion on how much residual amount can be determined as gastric emptiness, and its sensitivity is poor due to the wide range of determination of residual amount.^[14–16]^ Meanwhile, despite various drugs and treatment attempts, people are still often forced to discontinue enteral feeding due to severe EN intolerance.^[17–22]^

The gut microbiota plays an important role in maintaining the intestinal function, and the changes in the structure and composition of gut microbiota under stress state may be related to EN intolerance. The intestinal micro-ecosystem is known as the “second genome” and “second brain” of the human body. Gut microbiota and host depend on and restrict each other to maintain the normal physiological and health status. The composition of gut microbiota reflects the intestinal microenvironment and intestinal function of the host.^[23]^ In recent years, with the development of 16S ribosomal RNA (rRNA) and metagenomic sequencing technology, the role of gut microbiota in the occurrence of human diseases has been increasingly studied. In the field of intensive care medicine, a series of studies have been conducted on the relationship between gut microbiota and bacterial translocation, enteric infection, antibiotic resistance, antibiotic-associated diarrhea, acute respiratory distress syndrome, etc,^[24–30]^ yet there are few studies on gut microbiota and nutrient metabolism. Tremaroli et al^[31]^ studied the gut microbiota of patients after weight loss surgery and found that the composition of gut microbiota directly affects the amount of energy the host can obtain from the diet. On the other hand, the dietary structure also has a profound impact on gut microbiota. Both high-fat diet and obesity phenotype have been repeatedly proven to be associated with microbial migration.^[32]^

In the past, it was generally believed that the composition of EN should meet the metabolic needs of the host, without considering its effect on gut microbiota. It seems that this view has some limitations given that there exists an interaction between the host and the gut microbiota. As the composition of gut microbiota in ICU patients is closely related to the enteral nutrient uptake, the study on the relationship between gut microbiota and EN will shed light on the mechanism of EN intolerance and lay a foundation for the establishment of a new nutritional intervention strategy for ICU patients.

## 2 Methods

### 2.1 Study aim

The study aims to observe the effect of EN on the gut microbiota of ICU patients, and screen microbiological markers for predicting EN intolerance in ICU patients.

### 2.2 Study design and setting

**Figure 1:**
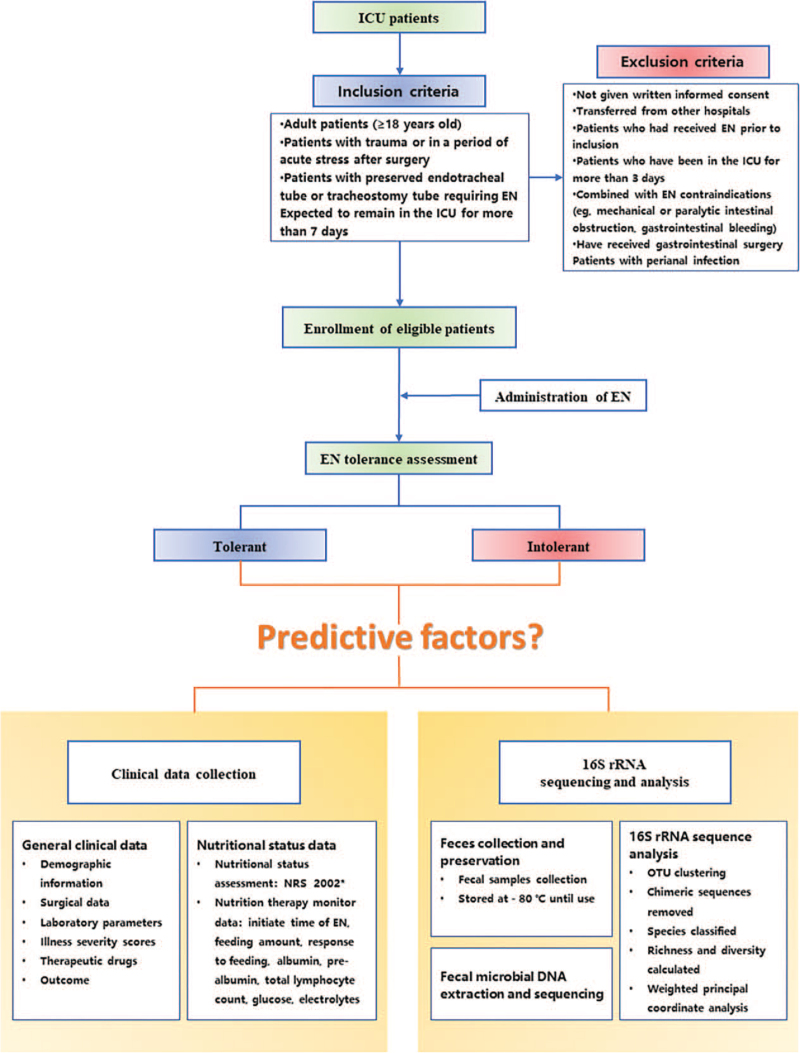
Flow diagram of this study. ^∗^NRS2002: is a nutritional risk screening tool that the ESPEN endorsed based on BMI, weight loss, and appetite as well as acute illness. BMI =  body mass index, EN = enteral nutrition, NRS 2002 = nutritional risk screening 2002, OTU = operational taxonomic units.

**Figure 2:**
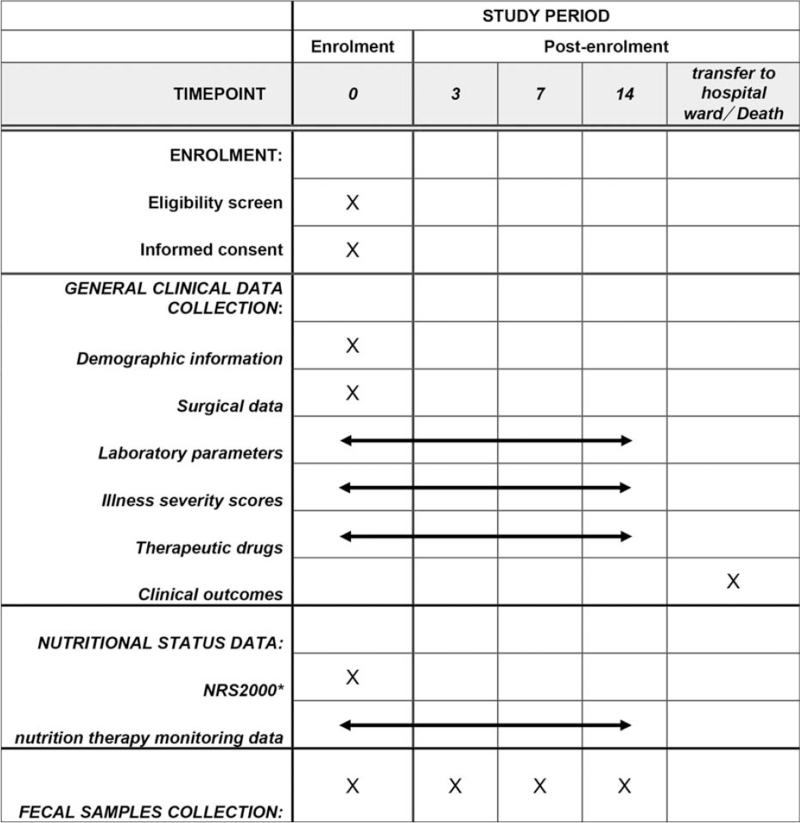
Schedule of enrolment and assessments according to SPIRIT.

The study protocol is designed to adhere to The SPIRIT (Standard Protocol Items: Recommendations for Interventional Trials) 2013 Statement.^[33]^ The present trial is a prospective cohort study conducted in a high-volume integrated ICU at the Beijing Tiantan Hospital, Capital Medical University, China. This study protocol was reviewed and approved by the Institutional Review Board of Beijing Tiantan Hospital, Capital Medical University (KY 2019-077-03). The study has been registered in the Chinese Clinical Trial Registry with the registration number ChiCTR2000031760. All eligible patients will be systematically proposed to participate in the study. After written informed consent has been obtained, patients will be enrolled. A flow diagram of this study is shown in Figure 1, and the schedule of the enrollment and assessments according to SPIRIT requirements is shown in Figure 2.

### 2.3 Study population

Patients will be included if they meet all the following criteria:

(1) Adult patients (≥18 years old)
(2) Patients in acute stress stage after neurosurgery or intracranial trauma
(3) Patients with tracheal intubation
(4) Patients who expected to receive treatment for >=7 days in ICU.

Patients will be excluded from the study if they meet any of the following exclusion criteria:

(1) Patients transferred from other hospitals
(2) Patients who had received EN before inclusion
(3) Patients who have been in the ICU for more than 3 days
(4) Patients who have contraindications to EN (e.g., mechanical, or paralytic intestinal obstruction, gastrointestinal bleeding; prone position patients)
(5) Patients who have undergone gastrostomy or jejunostomy
(6) Patients who have undergone rectal operation or with perianal infection.

### 2.4 Clinical data collection

We will obtain all clinical notes including general and nutritional specific data recorded in each patient's health record during their stay in the ICU.

#### 2.4.1 General clinical data

(1) Demographic information: ID, sex, age, weight (kg), height (cm), BMI calculation (kg/m^2^), comorbidities, smoking history, drinking history, medication history
(2) Surgical data: clinical diagnosis, surgical site, operative time, the amount of intraoperative blood loss and blood transfusion, intraoperative hypotension (mean arterial pressure <65 mm Hg) duration
(3) Laboratory parameters: blood count, platelet count, lymphocyte ratio, creatinine, liver tests (aspartate aminotransferase, alanine aminotransferase, gamma-glutamyl transferase, alkaline phosphatase), bilirubin
(4) Illness severity scores: Glasgow, Acute Physiology, and Chronic Health Evaluation II, and sequential organ failure assessment
(5) Therapeutic drugs: proton pump inhibitors, prokinetics, gastric mucosal protectors, coagulants, anticoagulants, antibiotics, vasopressors, anti-diarrhea medicine, cathartics, probiotics
(6) Clinical outcomes: duration of mechanical ventilation, length of ICU stay, length of hospital stay, mortality, infections

#### 2.4.2 Nutritional status data

(1) Nutritional status assessment: nutritional risk screening 2002^[34]^ (the ESPEN endorsed screening tool based on BMI, weight loss and appetite as well as acute illness)^[2]^
(2) Nutrition therapy monitoring data: initiate time of EN, feeding amount, response to feeding, albumin, pre-albumin, total lymphocyte count, glucose, electrolytes (potassium, magnesium, phosphate)

### 2.5 16S rRNA sequencing and analysis of structure and composition of gut microbiota

#### 2.5.1 Feces collection and preservation

Fecal samples will be collected on admission and days 3 and 7. For patients hospitalized in the ICU for longer than 14 days, an additional fecal sample will be collected on day 14. All fecal samples will be collected by inserting a sterile rectal swab into the anus 1 to 2 cm and rotating the swab 3 to 4 times. The swabs will be placed in a sterile Eppendorf tube and immediately stored in a refrigerator at −80°C until use.

#### 2.5.2 Fecal microbial DNA extraction and sequencing

Microbial DNA will be extracted using an OMEGA-soil DNA Kit (Omega Bio-tek, Norcross, GA). The V1–V3 regions of the 16S rRNA gene will be amplified by polymerase chain reaction with specific primers. After purified and quantified, the amplicons will be pooled and paired-end sequenced on an Illumina MiSeq platform (Illumina, San Diego, CA).

#### 2.5.3 16S rRNA sequence analysis

Operational taxonomic units (OTUs) will be clustered with a 97% similarity cutoff using UPARSE (a method for generating clusters from next-generation sequencing reads of marker genes, http://drive5.com/uparse/), and chimeric sequences will be identified and removed using UCHIME (an algorithm for detecting chimeric sequences, https://www.drive5.com/usearch/manual/uchime_algo.html). The taxonomy of the 16S rRNA gene sequence will be analyzed by RDP (Ribosomal Database Project) Classifier against the SILVA database (a web resource providing comprehensive, quality checked and regularly updated datasets of aligned small (16S/18S, SSU) and large subunit (23S/28S, LSU) ribosomal RNA (rRNA) sequences for all three domains of life (Bacteria, Archaea and Eukarya), https://www.arb-silva.de/). The alpha diversity will be calculated using Chao1, Shannon, and PD (Phylogenetic diversity) indexes based on the OTU profiles to estimate the richness and diversity of the samples. The weighted principal coordinate analysis will be performed based on UniFrac distances to measure community clustering.

### 2.6 Patient and public involvement

Patients and the general public were not involved in the design, enrollment, or implementation of the study. Patient care does not differ from the one usually carried out according to the recommendations. Study participants will be able to find the results of the study in scientific publications or conference presentations. They will not be directly informed.

### 2.7 Adverse outcomes

During the study, the researchers will ask about adverse events which may be related to the trial. If any adverse events occur, they will be noted in report form and reported in the publication.

### 2.8 Estimation of sample size

According to the preliminary survey results, the current EN intolerance rate in the ICU of Beijing Tiantan Hospital is 57%. It is expected that the EN intolerance rate will decrease to 40% after adjusting the EN feeding scheme according to the composition of intestinal flora. The 2-sided type I error rate is 5%, and the power is 90%. In consideration of 10% attrition, the final sample size is ∼100 (89/0.9) cases.

### 2.9 Statistical analysis

The continuous data will be presented as mean ± standard deviation or median (interquartile interval) according to a statistical distribution. The normality will be studied by the Shapiro–Wilk test. The categorical variables will be presented with the number of patients and percentages. To determine prognostic factors for EN intolerance, we will start by univariate analyses using the log-rank test for categorical variables and by the Cox model for continuous parameters. Then, a multivariable analysis will be performed using the Cox proportional-hazards model. The covariates will be determined according to univariate results and their clinical relevance. Kruskal–Wallis rank-sum test will be used to compare species abundance in the gut microbiota data. All tests are bilateral, and *P* < .05 is considered statistically significant. Data related to the gut microbiota will be statistically analyzed by R software, and other data performed by SPSS23.0 (SPSS, Inc, Chicago, IL) software.

## 3 Discussion

Nutritional status is closely related to the prognosis of critically ill patients, and nutrition support is an important part of the treatment for these patients. However, at present, the focuses of nutritional therapy on ICU patients are still restricted to the amount of energy to provide, the route to choose for energy supply, the feeding tube placement, and nitrogen balance, etc. What is ignored is that how these exogenous nutrients interact with the host and what effect will they take.

Gut microbiota is a complex ecosystem in the host, and changes in gut microbiota due to environmental impacts can affect the host's overall health. Particularly, critical illness is considered one of the major environmental factors that can affect the stability of the normal intestinal environment. Recent studies reported that when patients were in the ICU, the alpha-diversity of their gut microbiota declined and the gut microbiota was characterized by depletion of potential “health-promoting” commensal genus (e.g., *Faecalibacterium*, *Ruminococcus*, or *Pseudobutyrivibrio*) and overgrowth of pathobionts such as *Enterococcus*, *Escherichia*, *Staphylococcus*, Enterobacteriaceae, and *Pseudomonas*.^[29,35–37]^ Since more than 60% of ICU patients are forced to discontinue EN due to EN intolerance, and there is still no effective treatment method, it is urgent to seek new treatment methods to improve the situation. Exploring the composition and function of gut microbiota in ICU patients receiving EN may help to clarify the causes of EN intolerance and develop better strategies for EN treatment.

In recent years, researchers have proposed that particular gut microbiota signatures could be used to predict the occurrence, development, or clinical outcomes of certain diseases. Agudelo-Ochoa et al^[38]^ found that the gut microbiota of ICU patients with sepsis has an increased abundance of microbes tightly associated with inflammation and the abundance of pathogenic species, such as *Enterococcus spp*., was differentially increased in sepsis patients who died. Reitmeier et al^[39]^ demonstrated in 1976 subjects of a German population cohort (KORA, a prospective cohort in the region of Augsburg designed to understand the role of genetic, lifestyle, and environmental factors in disease progression including metabolic diseases) that specific microbiota members show 24-hour oscillations in their relative abundance and identified 13 taxa with disrupted rhythmicity in type 2 diabetes. Tang et al^[40]^ identified a microbiome signature containing 21 OTUs that can potentially predict postoperative Hirschsprung-associated enterocolitis with ∼85% accuracy.

The overarching objective of this study is to establish a predicting and monitoring system for EN intolerance in ICU patients based on gut microbiota. Focusing on the relationship between gut microbiota and the host intestinal environment, this project will study the structure and composition of gut microbiota in ICU patients using 16S rRNA sequencing technology, taking patients with trauma and postoperative acute stress as the research subjects. The results of this study will help us to find out the potential probiotics as dietary supplements to improve EN intolerance. The limitation of this study is the single-center design, which may potentially cause recruitment biases.

## Author contributions

**Conceptualization:** Yangyang Guo, Guangzhi Shi.

**Data curation:** Yangyang Guo, Guangzhi Shi.

**Formal analysis:** Yangyang Guo, Jindong Zhang.

**Funding acquisition:** Yangyang Guo, Jindong Zhang.

**Investigation:** Yangyang Guo, Ming Xu, Jindong Zhang.

**Methodology:** Yangyang Guo.

**Project administration:** Ming Xu.

**Software:** Yangyang Guo, Jindong Zhang.

**Supervision:** Guangzhi Shi.

**Validation:** Yangyang Guo, Ming Xu, Guangzhi Shi, Jindong Zhang.

**Writing – original draft:** Yangyang Guo.

**Writing – review & editing:** Jindong Zhang.
